# Comparative analyses of proteins from *Haemophilus influenzae* biofilm and planktonic populations using metabolic labeling and mass spectrometry

**DOI:** 10.1186/s12866-014-0329-9

**Published:** 2014-12-31

**Authors:** Deborah MB Post, Jason M Held, Margaret R Ketterer, Nancy J Phillips, Alexandria Sahu, Michael A Apicella, Bradford W Gibson

**Affiliations:** The Buck Institute for Research on Aging, Novato, CA 94945 USA; Washington University School of Medicine, St. Louis, MO 63110 USA; The University of Iowa, Iowa City, IA 52242 USA; The University of California San Francisco, San Francisco, CA 94143 USA

**Keywords:** Non-typeable *Haemophilus influenzae*, Metabolic labeling, Biofilms, Mass spectrometry

## Abstract

**Background:**

Non-typeable *H. influenzae* (NT*Hi*) is a nasopharyngeal commensal that can become an opportunistic pathogen causing infections such as otitis media, pneumonia, and bronchitis. NT*Hi* is known to form biofilms. Resistance of bacterial biofilms to clearance by host defense mechanisms and antibiotic treatments is well-established. In the current study, we used stable isotope labeling by amino acids in cell culture (SILAC) to compare the proteomic profiles of NT*Hi* biofilm and planktonic organisms. Duplicate continuous-flow growth chambers containing defined media with either “light” (L) isoleucine or “heavy” (H) ^13^C_6_-labeled isoleucine were used to grow planktonic (L) and biofilm (H) samples, respectively. Bacteria were removed from the chambers, mixed based on weight, and protein extracts were generated. Liquid chromatography-mass spectrometry (LC-MS) was performed on the tryptic peptides and 814 unique proteins were identified with 99% confidence.

**Results:**

Comparisons of the NT*Hi* biofilm to planktonic samples demonstrated that 127 proteins showed differential expression with p-values ≤0.05. Pathway analysis demonstrated that proteins involved in energy metabolism, protein synthesis, and purine, pyrimidine, nucleoside, and nucleotide processes showed a general trend of downregulation in the biofilm compared to planktonic organisms. Conversely, proteins involved in transcription, DNA metabolism, and fatty acid and phospholipid metabolism showed a general trend of upregulation under biofilm conditions. Selected reaction monitoring (SRM)-MS was used to validate a subset of these proteins; among these were aerobic respiration control protein ArcA, NAD nucleotidase and heme-binding protein A.

**Conclusions:**

The present proteomic study indicates that the NT*Hi* biofilm exists in a semi-dormant state with decreased energy metabolism and protein synthesis yet is still capable of managing oxidative stress and in acquiring necessary cofactors important for biofilm survival.

**Electronic supplementary material:**

The online version of this article (doi:10.1186/s12866-014-0329-9) contains supplementary material, which is available to authorized users.

## Background

Non-typeable *Haemophilus influenzae* (NT*Hi*) is a Gram-negative organism that is a typical component of the normal human nasopharyngeal flora. Under certain conditions, this bacterium can also be an opportunistic pathogen causing upper and lower respiratory tract infections such as otitis media, pneumonia, and bronchitis [1-4]. Infants, young children, the elderly, and persons suffering from chronic obstructive pulmonary disease (COPD) are the most common populations impacted by these opportunistic infections [1,4,5].

*H. influenzae* is able to form biofilms under both *in vitro* and *in vivo* conditions [6-12]. These biofilms are bacterial communities that exhibit characteristics which differentiate them from planktonic organisms [13]. One of these unique biofilm characteristics is resistance to clearance by antibiotics and the immune system [13-16]. This resistance is most likely responsible for the recurrent infections sometimes seen with *H. influenzae* [17-19]. The generation of a biofilm matrix is also a typical characteristic of a biofilm [20]. The *H. influenzae* biofilm matrix has been shown to consist of a number of components including double-stranded DNA, type IV pilin protein and sialylated lipooligosaccharide (LOS) [6,10,21-25]. The biofilm matrix typically generates an oxygen depleted environment within the biofilm and at its base. Nutrients are transported from the outer periphery of the matrix to its lower layers through nutrient channels. This matrix most likely plays a role in resistance to host defenses and antimicrobial therapies.

Several studies have been conducted to examine the components of the *H. influenzae* biofilm matrix, but to date no study has been initiated to examine the differences between the proteomic profiles of *H. influenzae* biofilm and planktonic bacteria. In the present study we utilize stable isotope labeling of amino acids in cell culture (SILAC) combined with mass spectrometry [26-28] to compare proteins expressed in biofilm and planktonic bacteria. Our group and others have successfully utilized SILAC to compare bacterial populations grown under differing conditions [29-33]. SILAC incorporates an amino acid labeled with a heavy stable isotope into one population; following proteolytic digestion, any peptides containing the labeled amino acid are then shifted by a specific mass. This mass shift allows one to directly compare the protein levels of the two test populations upon mixing and subsequent LC/MS analysis. In the present study, ^13^C_6_-isoleucine was incorporated into biofilm-grown *H. influenzae*. These bacteria were compared to planktonic *H. influenzae* grown in normal isoleucine. These populations were subsequently compared using both MS/MS analyses as well as selected reaction monitoring (SRM)-MS analyses. Mass spectrometry-based proetomic analyses enabled us to generate a list of proteins of interest. Targeted quantitative analyses of a subset of these proteins were subsequently performed using SRM-MS.

## Methods

### Bacterial strains and growth conditions

**Figure 1:**
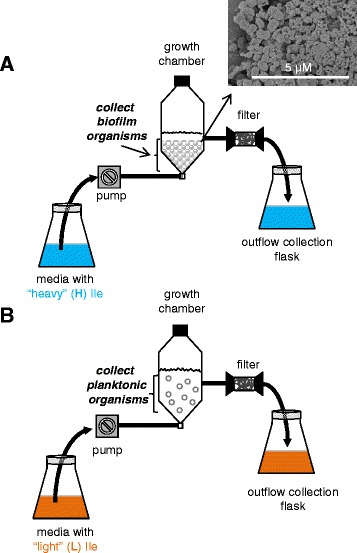
**Schematic illustrations of the continuous-flow growth chambers used to grow the (A) biofilm and (B) planktonic organisms.** The biofilm (“B”) organisms were grown with “heavy” ^13^C_6_-Isoleucine and the planktonic organisms (“P”) were grown with unlabeled or “light” isoleucine. The inserted micrograph in panel **(A)** is a scanning electron microscopy image showing a typical biofilm from these growth chambers.

The previously isolated non-typeable *H. influenzae* strain 2019 was utilized for all studies [34]. Bacteria were grown on agar culture plates made with defined RPMI medium, containing either normal isoleucine (planktonic cultures) or with heavy ^13^C_6_-labeled isoleucine (biofilm cultures) (Cambridge Isotope Labs, Andover, MA). Inoculation samples were grown in liquid defined RPMI medium, containing the same isoleucine as the plate cultures. Biofilm and planktonic organisms were grown in specially designed biofilm growth chambers, which were set-up as previously described except glass beads were used instead of granite pieces [35]. Two large growth chambers were set-up using the appropriate form of isoleucine, and after three days of growth, the planktonic bacteria (“light”) were collected from the liquid media from one chamber, and the biofilm growth (“heavy”) was collected from the glass beads of the other chamber (Figure 1).

Confirmation that the bacteria grew as a biofilm on the glass beads was obtained using scanning electron microscopy (Figure 1). Samples were prepared by lifting the beads carefully out of the chamber and gently immersing them in a solution of 1% OsO_4_ in perfluorocarbon (Fluorinert FC-72 from 3 M Specialty Fluids, St. Paul, MN). The samples were held stationary in this solution at room temperature for 3 h, and then washed in 3 changes of perfluorocarbon, followed by dehydration in 100% ethanol and final clearance in hexamethyl-disilazane (Polysciences, Inc., Warrington, PA). The beads were mounted on Al stubs, sputter-coated with Au-Pd, and viewed with the Hitachi S4800 scanning electron microscope, housed at the University of Iowa Central Microscopy Research Facility, at an accelerating voltage of 2.0 kV.

### Protein sample preparation

**Figure 2:**
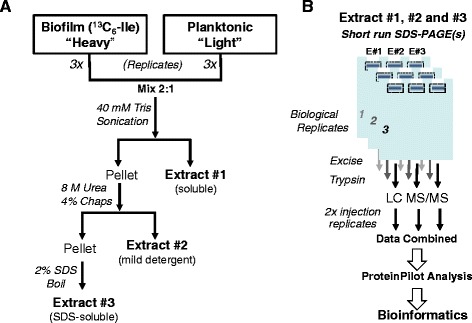
**Workflow of sample preparation and analyses. (A)** Three sequential extracts were generated in 3 biological replicates and **(B)** were further separated by SDS-PAGE to enhance protein identification and quantification coverage. The data obtained from the LC/MS experiments (2 injections replicates per sample) were combined for each biological replicate and searched using Protein Pilot.

Biofilm and planktonic organisms were lyophilized to dryness, weighed, and combined at a ratio to yield equivalent levels of total protein (2:1, biofilm:planktonic). As can be seen in Figure 2, sequential protein extracts were generated using the Bio-Rad Ready Prep Sequential extraction kit (Bio-Rad, Hercules, CA) which generates three sequential protein fractions: soluble, urea-soluble, and SDS-soluble. Bacteria were suspended in 40 mM Tris, sonicated five times for 30 sec each with 1 min rest intervals on ice, and centrifuged at 16,000 × *g* for 3 min. The supernatant of this was designated “extract one”, the soluble extract. The pellet was suspended in 8 M urea, 4% CHAPS, and 12 mM tributyl phosphine and was subsequently centrifuged. The supernatant from this was designated “extract two” (urea soluble fraction). The insoluble pellet was washed twice, suspended in 2% SDS, boiled for 10 min, and designated “extract three” (SDS soluble fraction). The amount of protein in each extract was determined and 60 μg of protein from each extract was loaded into separate lanes of a 4-12% SDS-PAGE gel (NuPage, Life Technologies, Carlsbad, CA). Samples were run 2 cm into the gel and visualized with Simply Blue Safe Stain (Life Technologies), and manual in-gel trypsin digestion was performed. Proteins were excised (5–6 bands excised per extract) from the gels and destained and dehydrated with acetonitrile (50% acetonitrile/ 25 mM NH_4_HCO_3_). Proteins were reduced with 10 mM DTT in 25 mM NH_4_HCO_3_ for 1 h at 56°C and subsequently alkylated with 55 mM iodoacetamide for 45 min at room temperature. Proteins were digested with trypsin using a trypsin concentration of 1:20 (trypsin:protein) for 16–18 h at 37°C. Peptides were first extracted with water and subsequently with 50% acetonitrile/5% formic acid. Samples were concentrated under vacuum to a final volume of 5–10 μl. Approximately 5–10 μl of 5% acetonitrile/0.5% formic acid was added to the samples to achieve a final volume of 15 μl.

### MALDI-TOF analyses

To assess proper incorporation of the heavy-labeled isoleucine, peptide samples were initially evaluated by matrix-assisted laser desorption ionization time-of-flight (MALDI-TOF) mass spectrometry on a Voyager DE-STR mass spectrometer (AB Sciex, Concord, Canada) operating in the positive-ion reflectron mode under delayed extraction conditions: 200 ns delay time, with a grid voltage of 66.5% of full acceleration voltage (25 kV). Samples were mixed 1:1 with α-cyano-4-hydroxycinnamic acid (CHCA) matrix and spotted onto a stainless steel target. Mass spectra were acquired, averaged (typically 100 laser shots), and externally calibrated with a standard peptide mixture consisting of angiotensin I, and ACTH fragments 1–17, 18–39, and 7–38 (Bachem, Torrance, CA).

### Nano-LC-ESI MS/MS analyses

To compare the relative expression levels of proteins between the biofilm and planktonic populations, peptides generated after proteolytic digestion of all three extracts were analyzed by reverse-phase nano-HPLC-MS/MS using an Eksigent nano-LC 2D HPLC system that was directly connected to a quadropole-TOF mass spectrometer (QSTAR Elite, AB Sciex, Concord, Canada). Peptides were loaded onto a guard column (C18 Acclaim PepMap100, 300 μm I.D. × 5 mm, 5 μm particle size, 100 Å pore size, Dionex, Sunnyvale, CA) and washed with loading solvent for 10 min (98% solvent A, 2% solvent B, where solvent A is 0.1% formic acid in 98% H_2_O, 2% acetonitrile and solvent B is 0.1% formic acid in 98% acetonitrile, 2% H_2_O) at a flow rate of 20 μl/min. Samples were then transferred onto a C18 nano-HPLC analytical column (C18 Acclaim PepMap100, 75 μm I.D. × 15 cm, 3 μm particle size, 100 Å pore size, Dionex) and eluted at a flow rate of 300 nL/min using either an ~2 h (extracts 2 and 3) or an ~3 h gradient (extract 1). The gradient for the 2 h runs was: 2-40% solvent B in A (from 0–60 min), 40-90% solvent B in A (from 60–75 min) and 90% solvent B in A (from 74–85 min), for a total of 120 min including column equilibration. The gradient for the 3 h runs was: 98% solvent A in B (from 0–5 min), 2-40% solvent B in A (from 5–125 min), 40-90% solvent B in A (from 125–140 min), and 90% solvent B in A (from 140–149 min) for a total of 180 min including column equilibration. Electrospray ionization mass spectra (ESI-MS) and tandem mass spectra (ESI-MS/MS) were recorded in positive-ion mode with a resolution of 12,000-15,000 FWHM. An ion spray voltage of 2300 V, curtain gas of 20 psi, ion source gas of 17 psi, and an interface heater temperature of 100°C were used for acquisition of the data. For collision induced dissociation tandem mass spectrometry (CID-MS/MS), the mass window for precursor ion selection of the quadrupole mass analyzer was set to ± 1 *m/z*. Information dependent acquisition was utilized for MS/MS data acquisition. Two injection replicates were run for all samples.

### Data analyses of ESI-MS and MS/MS data

All files generated from the QSTAR Elite were analyzed using Protein Pilot 4.1 (revision 460) running the Paragon Algorithm 4.0.0.0, 459 (Applied Biosystems) [36] with a custom database consisting of the predicted protein sequences from *H. influenzae* strain 86-028NP [37]. Search parameters included: sample type was defined as SILAC (Ile + 6), Cys alkylation method was iodoacetamide, instrument type was QSTAR ESI, and trypsin was the proteolytic enzyme. A “thorough search” was then performed with both ‘quantitate’ and ‘bias correction’ features selected. Imperfect 1:1 mixing of the samples was corrected for in the dataset by this bias correction factor, so reported H:L ratios were normalized allowing direct comparisons to be made between datasets. False Discovery Rate Analysis (FDR) was performed using the default setting for the “detected protein threshold [Ununsed ProtScore (Conf)]” at >0.05 (10.0%). The Proteomics System Performance Evaluation Pipeline (PSPEP) tool was used to generate the FDR analyses using a concatenated forward and reverse decoy database to search the data. The output from this analysis shows the FDR at the spectral, peptide and protein levels [38]. In the present study, we included in our datasets only proteins with an “Unused ProtScore” of ≥2.0, which corresponds to a protein confidence cut-off threshold of 99%. At this protein confidence threshold, the protein global false discovery rate (FDR) from fit of the data was 0.0048% or lower in all of the proteomics datasets. The default setting of “Confidence Percent Threshold for Including Self In Quant” within the “ProteinPilot.exe.config” file was changed from 15% to 50%, thus only peptides with a confidence score of at least 50% were used to derive the H:L ratios for protein quantitation.

All of the output ratios for the quantitative SILAC results were expressed as H:L or biofilm:planktonic (B:P). Only quantified proteins with at least two peptides identified and overall p-values ≤0.05 were included in our final datasets of the differentially expressed proteins, a fold-change threshold was not applied to the data. Predicted protein roles were determined using the role identification tool from the J. Craig Venter Institute Comprehensive Microbial Resource website (http://cmr.jcvi.org/cgi-bin/CMR/CmrHomePage.cgi) [39]. In a few cases this search generated a predicted protein role as “unknown”. These proteins were further investigated using the “protein knowledgebase (UniProtKB)” from the UniProt website (http://www.uniprot.org/) [40], and if further information was obtained, the role was changed appropriately. Protein localization was predicted using the web-based version of the PSORTb tool version 3.0.2 (http://www.psort.org/psortb/index.html) [41].

### LC-SRM/MS

To increase selectivity and sensitivity of our differential measurements, a select group of peptides were targeted for analysis by nano-LC-SRM/MS on a 4000 QTRAP hybrid triple quadrupole/linear ion-trap mass spectrometer (AB Sciex). Chromatography was performed using a NanoLC-2D LC system (Eksigent, Dublin, CA) with solution A (0.1% formic acid) and solution B (90% acetonitrile in 0.1% formic acid). Samples were loaded onto a trap column at 5 μl/min onto a 5 mm × 300 μm reversed phase Dionex C18 trap column (5 μm, 100 Å) for 10 min and eluted at 300 nL/min with a gradient of 2-70% solution B over 32 min using an in-house packed Integrafrit analytical column (75 μm I. D., New Objective, Woburn, MA) with 10–12 cm of ReproSil-Pur C18-AQ 3 μm reversed phase resin (Dr. Maisch GmbH, Germany). Peptides were ionized using a PicoTip emitter (75 μm, 15 μm tip, New Objective). Data acquisition was performed using Analyst 1.5 (AB Sciex) with an ion spray voltage of 2450 V, curtain gas of 10 psi, nebulizer gas of 20 psi, and an interface heater temperature of 150°C. To develop SRM transitions, data files from the QSTAR Elite runs were imported into the publicly available software Skyline. A detailed description of the software is described elsewhere [42,43]. SRM transitions were selected from proteins predicted to have differential expression between the biofilm and planktonic samples. In total, 368 transitions were analyzed in a single LC-SRM/MS analysis with identical dwell times, collision energies, declustering potentials and collision cell exit potentials for each pair of labeled and unlabeled peptides. All of the soluble extract fractions from all of the biological replicates were analyzed by SRM, except one fraction from experiment #2 because the sample was lost due to technical difficulties. Skyline software was utilized for data analyses. The data from the strongest fraction and the best transition was extracted from Skyline and analyzed. Observed peptides needed to have relatively consistent retention times across the various biological replicates and between the “heavy” and “light” versions of the peptide and they also needed to have a signal to noise ratio of at least 2:1 to be included in our dataset. The observed bias factor for each biological replicate was applied to the data extracted from the best transition. Average ratios were calculated by converting ratios to log2, averaging and then converting these results back to normal numbers.

### Data accession

All raw data associated with this manuscript may be downloaded from the massIVE ftp site at ftp://MSV000078838@massive.ucsd.edu [44].

## Results

### Sample preparation

RPMI media supplemented with either normal isoleucine (“light”) or ^13^C_6_-isoleucine (“heavy”) was used to culture the planktonic and biofilm organisms, respectively. Both samples were prepared utilizing large growth chambers as shown in Figure 1. The presence of a typical biofilm on the glass beads was confirmed using scanning electron microscopy. For sample collection, biofilm organisms were washed from the glass beads, rinsed with PBS, and lyophilized to dryness. Planktonic organisms were collected from the supernatant of the biofilm chamber, rinsed with PBS, and lyophilized to dryness. The lyophilized samples were initially mixed 1:1 based on dry weight. Proteolytic peptides from this 1:1 sample were generated as described above, and subsequently analyzed using LC-MS. The bias factor feature of Protein Pilot, which measures how accurate the sample mixing is to 1:1, when all peptides containing isoleucine are compared, was utilized to determine how close to a 1:1 protein mix was actually obtained. The derived bias factor from this initial dataset was 0.57 (biofilm:planktonic), indicating that our samples contained twice as much proteins from the planktonic organisms than from the biofilm. This disproportionate mixing was expected as it is known that biofilm organisms generate an extracellular matrix which the planktonic organisms do not make. Therefore, in subsequent experiments we mixed the samples 2:1 (biofilm:planktonic) by weight, to yield a more comparable level of proteins between the two states.

To determine the efficiency of the label incorporation into the biofilm samples, equivalent amounts of biofilm and planktonic samples were separated by 1-D SDS-PAGE, individual bands extracted, trypsin digestion performed, and the samples analyzed by MALDI-TOF. The ^13^C_6_-isoleucine utilized to label the biofilm organisms was purported by the manufacturer to have 98% labeling efficiency. The isotopic distributions from two representative isoleucine-containing peptides from two different proteins were used to determine incorporation efficiency. The peak intensities from 10 different spectra, of each peptide, were averaged and compared to MS-Isotope’s (Protein Prospector web-based tool, University of California, San Francisco Mass Spectrometry Facility) predicted isotopic distribution of the peptide. These data showed that the predicted incorporation of the “heavy” isoleucine was 97.5%. These data demonstrated that the ^13^C_6_-Ile was properly and efficiently incorporated into the biofilm proteins. In addition, peptides without isoleucine showed no evidence of altered isotopic composition, demonstrating that the label had not been catabolized into other amino acids.

### LC-ESI-MS/MS data

To reduce the complexity of the mixed samples and increase coverage, three extracts were generated based on protein solubility (Figure 2). Each extract was briefly separated by SDS-PAGE, and then an in-gel trypsin digest was performed. Samples were prepared from three biological replicates and were analyzed by nano-LC-ESI MS/MS. Injection replicates were run for all samples. All of the data generated from a biological replicate was searched using Protein Pilot, which also determined the bias factor for each experiment. As stated previously, a perfect 1:1 mix of the “heavy” and “light” samples would generate a bias factor of 1. Protein Pilot determined that our experiments had bias factors of 0.986, 1.2277, and 1.0219 in biological replicates 1, 2, and 3 respectively. These data showed that we had achieved a reasonable mix of our biofilm and planktonic populations in each of the biological replicates, an important parameter for efficient matching and accurate quantitation of peptides from the two groups.

Overall 814 unique proteins were identified with 99% confidence and at least two peptides at 95% confidence, corresponding to ~45% of the total proteome (Additional file 1). In biological replicate one, 721 proteins were identified with 99% confidence and at least two peptides at 95% confidence (Additional file 2). Of these proteins, 33 proteins showed an upward trend in the biofilm samples while 35 proteins showed evidence of downregulation (Additional files 3 and 4). For biological replicate two, 723 proteins were identified with 99% confidence and at least two peptides at 95% confidence (Additional file 5), of these 11 appeared to be upregulated in the biofilm and 13 downregulated (Additional files 6 and 7). 668 proteins were identified with 99% confidence and at least two peptides at 95% confidence in biological replicate three (Additional file 8), with 21 proteins identified as being upregulated and 43 downregulated in the biofilm (Additional files 9 and 10). Comparison of the data from the three different biological replicates showed that 127 unique proteins were differentially expressed in the biofilm versus planktonic organisms (Table 1), corresponding to ~ 7% of the total proteome. Two proteins were found to be differentially expressed in all three biological replicates, while four were common between replicates #1 and #2, and twenty-three proteins were common between experiments #1 and #3. Four differentially expressed proteins were found to be in common between experiments #2 and #3. In total, we observed 27 proteins that were consistently up or down-regulated between two or more biological replicates (Table 1), and five proteins that showed conflicting trends between two biological replicates and were therefore not investigated further (Additional file 11).

**Table 1:** **Proteins Predicted to be up- or downregulated, by Protein Pilot, with p values ≤ 0.05**

		^**1**^ **Biofilm: Planktonic ratio**			
**Accession #**	**Protein name**	**Rep. 1**	**Rep. 2**	**Rep. 3**	^**2**^ **Localization**
**Amino acid biosynthesis**					
AAX87912.1	glutamine synthetase, GlnA	**1.71**		**1.88**	Cytoplasmic
AAX87713.1	ketol-acid reductoisomerase, IlvC			**1.26**	Cytoplasmic
AAX87519.1	D-3-phosphoglycerate dehydrogenase, SerA			0.54	Cytoplasmic
AAX87314.1	dihydrodipicolinate synthase, DapA	0.66			Cytoplasmic
**Biosynthesis of cofactors, prosthetic groups, and carriers**					
AAX87263.1	NAD nucleotidase, NadN	**2.47**	**1.62**	**1.23**	Periplasmic
AAX87920.1	octaprenyl-diphosphate synthase, IspB			0.71	Cytoplasmic
AAX87900.1	putative heme iron utilization protein			0.82	Cytoplasmic
**Cell envelope**					
AAX88031.1	transferrin-binding protein 1, Tbp1	**1.86**			OMP
AAX87746.1	VacJ lipoprotein	**1.58**			OMP
AAX87040.1	rod shape-determining protein MreB	**1.41**			Cytoplasmic
AAX88649.1	acylneuraminate cytidylyltransferase, SiaB			0.70	Cytoplasmic
AAX88164.1	Outer membrane protein P5		0.58		OMP
**Cellular processes**					
AAX88712.1	predicted periplasmic or secreted lipoprotein	**1.57**			Periplasmic
AAX87955.1	protective surface antigen D15	**1.44**			OMP
AAX88561.1	cell division protein MukB	**1.18**			Unknown
AAX88143.1	cell division protein FtsA		0.76		Cytoplasmic
AAX87967.1	catalase, HktE	0.76			Periplasmic
**Central intermediary metabolism**					
AAX87206.1	putative sialic acid transporter, TRAP-type C4-dicarboxylate transport system, periplasmic component, SiaP	0.67		0.63	Periplasmic
**DNA metabolism**					
AAX87735.1	DNA mismatch repair protein MutS	**2.91**			Cytoplasmic
AAX88107.1	DNA ligase, LigN		**2.26**		Cytoplasmic
AAX88664.1	transcription-repair coupling factor, Mfd	**1.48**			Cytoplasmic
AAX88280.1	DNA-binding protein H-NS homolog	**1.40**			Cytoplasmic
AAX88572.1	DNA topoisomerase I, TopA	**1.38**			Cytoplasmic
AAX87482.1	DNA-binding protein HU			**1.31**	Cytoplasmic
AAX87308.1	UvrABC system protein A		**1.28**		Cytoplasmic
AAX88029.1	DNA polymerase III, beta chain, DnaN			0.81	Cytoplasmic
**Energy metabolism**					
AAX87718.1	glycerophosphoryl diester phosphodiesterase precursor, Glp	**1.51**		**1.89**	Unknown
AAX88650.1	putative NAD(P)H nitroreductase, NsfB		**1.80**		Unknown
AAX87025.1	citrate lyase alpha chain, citF	**1.38**		**1.71**	Cytoplasmic
AAX88575.1	NAD(P) transhydrogenase subunit alpha, PntA	**1.43**		**1.62**	Cyto. Memb.
AAX88675.1	NADP-dependent malic enzyme, Mao2	**1.56**			Cytoplasmic
AAX88704.1	D-lactate dehydrogenase, Dld	**1.26**			Cyto. Memb.
AAX88019.1	6-phosphofructokinase, PfkA			0.52	Cytoplasmic
AAX87882.1	fumarate reductase flavoprotein subunit, FrdA		0.54		Cyto. Memb.
AAX88128.1	transaldolase, TalB			0.55	Unknown
AAX87573.1	fructose-bisphosphate aldolase, Fba	0.73		0.55	Cytoplasmic
AAX88202.1	acetate kinase, AckA	0.70		0.55	Cytoplasmic
AAX88159.1	thioredoxin reductase, TrxB			0.56	Cytoplasmic
AAX87856.1	phosphoenolpyruvate carboxykinase, PckA			0.57	Cytoplasmic
AAX88535.1	fumarate hydratase class II, FumC			0.59	Cytoplasmic
AAX87709.1	triosephosphate isomerase, TpiA			0.64	Cytoplasmic
AAX88580.1	1,4-alpha-glucan branching enzyme, GlgB			0.66	Cytoplasmic
AAX88208.1	malate dehydrogenase, Mdh	0.66			Unknown
AAX87248.1	flavodoxin, FldA	0.68			Cytoplasmic
AAX87583.1	aspartate ammonia-lyase, AspA			0.70	Cytoplasmic
AAX87004.1	glyceraldehyde 3-phosphate dehydrogenase, GapA		0.70	0.78	Cytoplasmic
AAX88293.1	pyruvate kinase, PykA			0.72	Cytoplasmic
AAX87574.1	phosphoglycerate kinase, Pgk	0.75			Cytoplasmic
AAX87971.1	enolase, Eno	0.77		0.81	Cytoplasmic
AAX88160.1	thioredoxin domain-containing protein, YbbN			0.78	Cytoplasmic
AAX87237.1	formate acetyltransferase, PflB			0.79	Cytoplasmic
**Fatty acid and phospholipid metabolism**					
AAX87797.1	glycerol-3-phosphate acyltransferase, PlsB	**1.50**			Cyto. Memb.
AAX87445.1	long-chain-fatty-acid--CoA ligase, LcfA	**1.60**			Cyto. Memb.
AAX88571.1	probable acyl carrier protein phosphodiesterase, AcpD	**1.50**			Cytoplasmic
**Hypothetical proteins**					
AAX87451.1	conserved hypothetical cupin superfamily metalloenzyme		**1.36**		Cytoplasmic
AAX88259.1	conserved hypothetical phosphate transport regulator			0.65	Cytoplasmic
AAX87425.1	conserved hypothetical protein		0.73		Unknown
AAX88050.1	conserved hypothetical protein	0.80			Cytoplasmic
**Protein fate**					
AAX87914.1	peptidase B, pepB			**1.52**	Cytoplasmic
AAX87592.1	60 kDa chaperonin, GroEL			**1.52**	Cytoplasmic
AAX87863.1	Xaa-Pro aminopeptidase, PepP			**1.51**	Cytoplasmic
AAX88683.1	chaperone protein DnaK			**1.42**	Cytoplasmic
AAX87793.1	protein-export protein SecB		**1.41**		Cytoplasmic
AAX87272.1	oligopeptidase A, PrlC	**1.37**	**1.28**		Cytoplasmic
AAX87706.1	aminoacyl-histidine dipeptidase, PepD	**1.32**			Cytoplasmic
AAX87591.1	10 kDa chaperonin, GroES	0.42			Cytoplasmic
AAX87652.1	peptidase E, PepE	0.52			Cytoplasmic
AAX87816.1	cell division protein FtsY			0.56	Cytoplasmic
AAX88211.1	thiol:disulfide interchange protein DsbC	0.58			Periplasmic
AAX87741.1	trigger factor, Tig			0.64	Cytoplasmic
AAX88760.1	cytosol aminopeptidase, PepA			0.72	Cytoplasmic
**Protein synthesis**					
AAX88424.1	phenylalanyl-tRNA synthetase beta chain, PheT			**2.11**	Cytoplasmic
AAX87782.1	prolyl-tRNA synthetase, ProS			**2.00**	Cytoplasmic
AAX87660.1	30S ribosomal protein S7, RpsG			**1.81**	Cytoplasmic
AAX87825.1	50S ribosomal protein L3, RplC			**1.53**	Cytoplasmic
AAX87593.1	50S ribosomal protein L9, RplI		**1.31**		Cytoplasmic
AAX88652.1	methionyl-tRNA synthetase, MetG		**1.29**		Cytoplasmic
AAX87177.1	Seryl-tRNA synthetase, SerS		**1.25**		Cytoplasmic
AAX87580.1	30S ribosomal protein S21, RpsU	0.49			Cytoplasmic
AAX87839.1	30S ribosomal protein S14, RpsN	0.47			Cytoplasmic
AAX87952.1	elongation factor Ts	0.59		0.58	Cytoplasmic
AAX87661.1	elongation factor G, FusA	0.61		0.76	Cytoplasmic
AAX87855.1	ribosome recycling factor, Frr	0.68	0.73	0.62	Cytoplasmic
AAX87080.1	cysteinyl-tRNA synthetase, CysS	0.62			Cytoplasmic
AAX88252.1	tyrosyl-tRNA synthetase, TyrS			0.63	Cytoplasmic
AAX87851.1	50S ribosomal protein L17, RplQ	0.66			Cytoplasmic
AAX87670.1	50S ribosomal protein L10, RplJ	0.67			Cytoplasmic
AAX87910.1	GTP-binding protein TypA/ BipA			0.68	Cyto. Memb.
AAX87836.1	50S ribosomal protein L14, RplN		0.71		Cytoplasmic
AAX87838.1	50S ribosomal protein L5, RplN	0.74			Cytoplasmic
AAX88285.1	arginyl-tRNA synthetase, ArgS	0.74			Cytoplasmic
AAX87448.1	predicted GTPase, probable translation factor			0.74	Cytoplasmic
AAX87662.1	elongation factor Tu, TufB		0.80	0.81	Cytoplasmic
AAX88543.1	valyl-tRNA synthetase, ValS			0.83	Cytoplasmic
AAX87376.1	aspartyl-tRNA synthetase, AspS			0.85	Cytoplasmic
**Purines, pyrimidines, nucleosides, and nucleotides processes**					
AAX87406.1	adenylate kinase, Adk	0.55		0.66	Cytoplasmic
AAX88253.1	Ribose-phosphate pyrophosphokinase, PrsA	0.57		0.80	Cytoplasmic
AAX87655.1	2’,3’-cyclic-nucleotide 2’-phosphodiesterase, CpdB	0.78		0.61	Periplasmic
AAX88085.1	CTP synthase, PyrG		0.62		Cytoplasmic
AAX87567.1	Purine nucleoside phosphorylase, DeoD			0.65	Cytoplasmic
AAX87928.1	Serine hydroxymethyltransferase, GlyA	0.75			Cytoplasmic
**Regulatory functions**					
AAX87211.1	HflK	**1.36**			Cytoplasmic
AAX87210.1	HflC	**1.22**			Cytoplasmic
AAX87923.1	aerobic respiration control protein ArcA	0.37		0.35	Cytoplasmic
**Transcription**					
AAX88453.1	transcription elongation factor GreA	**1.60**			Cytoplasmic
AAX88611.1	transcription elongation protein NusA			**1.44**	Cytoplasmic
AAX87564.1	DNA-directed RNA polymerase beta chain, RpoB	**1.17**		**1.32**	Cytoplasmic
AAX87290.1	polyribonucleotide nucleotidyltransferase, Pnp	**1.17**		**1.18**	Cytoplasmic
AAX87467.1	ribonuclease E, Rne			**1.14**	Cytoplasmic
AAX87292.1	Cold-shock DEAD-box protein A homolog, DeaD		0.52		Cytoplasmic
**Transport and binding proteins**					
AAX88593.1	spermidine/putrescine-binding periplasmic protein 1 precursor, PotD1			**2.04**	Periplasmic
AAX88216.1	putative L-lactate permease	**1.96**		**1.68**	Cyto. Memb.
AAX87899.1	heme-binding protein A, HbpA	**1.73**			Periplasmic
AAX87717.1	glycerol-3-phosphate transporter, GlpT	**1.50**			Cyto. Memb.
AAX87322.1	heme/hemopexin-binding protein B, HxuB	**1.45**			OMP
AAX88215.1	heme utilization protein, Hup	**1.37**			OMP
AAX88590.1	spermidine/putrescine transport ATP-binding protein, PotA		**1.34**		Cyto. Memb.
AAX87417.1	putative periplasmic chelated iron binding protein, HfeA	**1.31**			Periplasmic
AAX88729.1	predicted regulator of cell morphogenesis and NO signaling	0.77		0.39	Cytoplasmic
AAX88749.1	molybdate-binding periplasmic protein, ModA	0.46	0.69		Periplasmic
AAX87869.1	D-galactose-binding periplasmic protein precursor, MglB	0.64		0.59	Periplasmic
AAX88127.1	periplasmic oligopeptide-binding protein, OppA	0.71		0.61	Periplasmic
AAX87555.1	Ribose-binding periplasmic protein, RbsB		0.68		Periplasmic
AAX88552.1	phosphate-binding periplasmic protein precursor PstS	0.72		0.70	Periplasmic
AAX88551.1	ferritin like protein 1, FtnA	0.77			Cytoplasmic
AAX87184.1	high-affinity zinc uptake system protein ZnuA	0.77			Periplasmic
AAX87223.1	Na(+)-translocating NADH-quinone reductase subunit A, NqrA		0.84		Cytoplasmic

^1^Bolded ratios show proteins predicted to be upregulated in the biofilm. Non-bolded ratios are proteins predicted to be downregulated in the biofilm.

^2^Localization determined using PSORTb program. OMP = outer membrane protein, Cyto. Memb. = cytoplasmic membrane protein.

**Figure 3:**
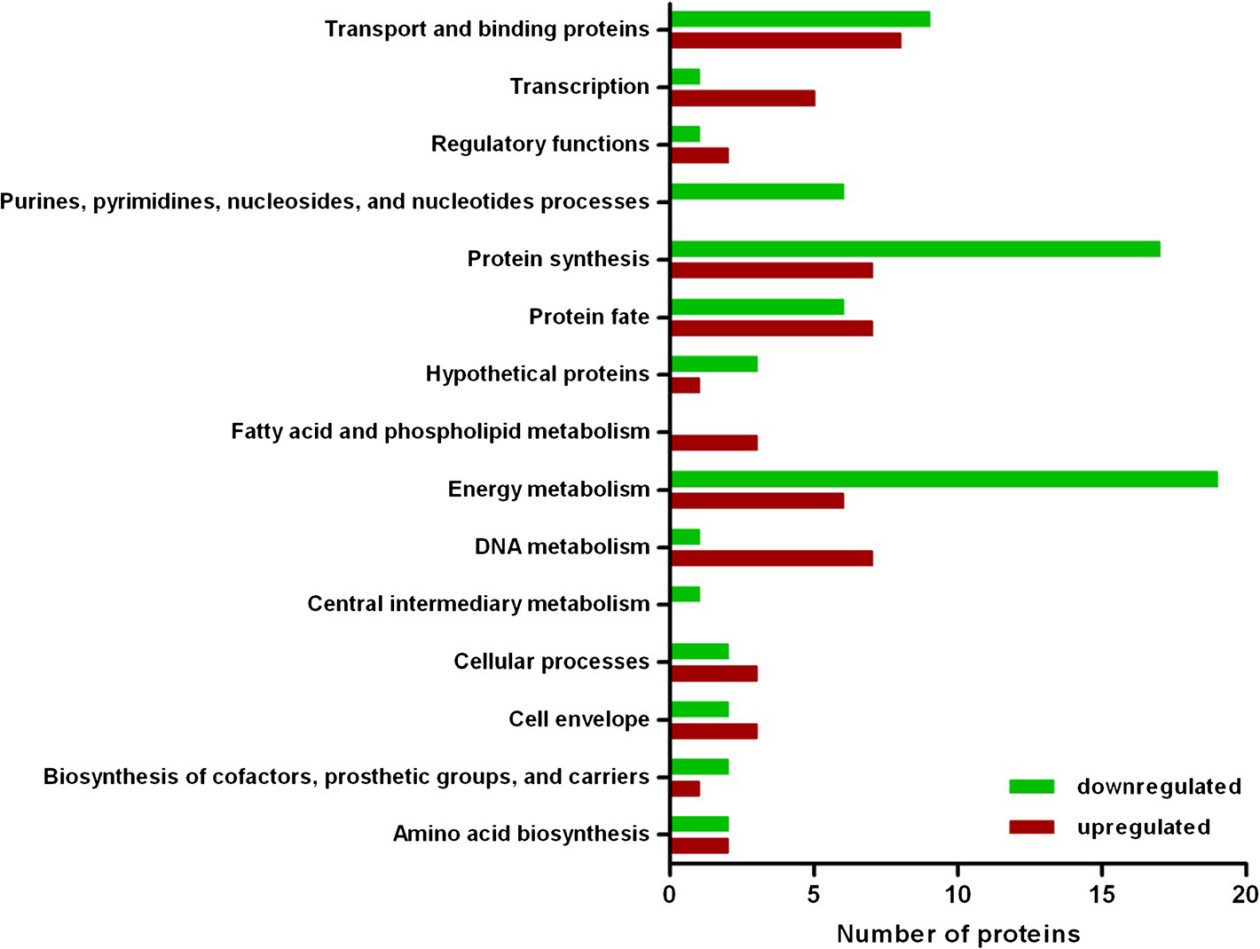
**Plot of functional classifications of proteins predicted to be up- or downregulated in the biofilm compared to the planktonic organisms.** The functional roles of the 127 proteins were determined using the role identification tool from the J. Craig Venter Institute Comprehensive Microbial Resource website (http://cmr.jcvi.org/cgi-bin/CMR/CmrHomePage.cgi) or the protein knowledgebase “(UniProtKB)” from the UniProt website (http://www.uniprot.org/). The proteins plotted had quantification ratios with p values ≤ 0.05.

When the functional roles of the 127 proteins that showed differential expression between the biofilm samples and the planktonic organisms were examined, a number of key processes were identified (Figure 3). A trend towards downregulation was seen in proteins involved in energy metabolism, protein synthesis, and purine, pyrimidine, nucleoside, and nucleotide processes. Analysis of the data showed that 76% of the proteins (19/25 proteins) involved in energy metabolism showed a downward trend in the biofilm population. Further metabolic pathway analysis was performed on the proteins involved in energy metabolism using the KEGG website [45]. These analyses showed that a number of these proteins could be mapped to the pyruvate metabolism, glycolysis and pentose phosphate pathways (Additional files 12, 13 and 14). These data also showed that 71% (17/24 proteins) of the proteins involved in protein synthesis and identified as having differential protein expression were downregulated. All six of the proteins classified as being involved in purine, pyrimidine, nucleoside, and nucleotide processes, which were identified as being differential expressed in this study, showed a trend towards downregulation. Conversely, proteins involved in transcription, DNA metabolism, and fatty acid and phospholipid metabolism appeared to have an increased expression level in the biofilm. 83.3% (5/6) of the proteins identified as being involved in transcription were upregulated in the biofilm. All three of the proteins classified in fatty acid and phospholipid metabolism showed higher expression levels in the biofilm bacteria than the planktonic organisms. 87.5% or 7/8 of the proteins classified as DNA metabolism proteins were upregulated in the biofilm bacteria.

### LC-SRM/MS data

**Figure 4:**
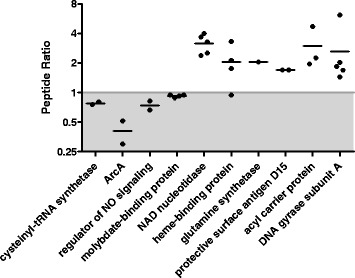
**Log scale plots of the peptide ratios of the Biofilm:Planktonic (B:P) data observed in the SRM-MS analyses.** Each dot represents the average of the B:P ratio for a distinct peptide. The line shows the overall average of the B:P data for each protein. Proteins whose average ratio showed downregulation in the biofilm appear in the grey shaded space of the plot. Proteins whose average ratio showed upregulation in the biofilm are plotted in the non-shaded area of the graph.

To obtain better sensitivity and accuracy in the relative quantitation of some of the more important proteins identified in the global SILAC analysis, SRM-MS was performed. Proteins were selected from the list of differentially expressed proteins observed in the ESI-MS/MS analyses; additionally proteins of potential biological significance were also targeted in these analyses. Peptides observed from this set of proteins were refined to include only peptides which contained no methionine or cysteine residues, no missed cleavages, no N-terminal glutamine or glutamic acid, and no adjacent tryptic sites. Nineteen proteins with at least one peptide which met the above criteria were analyzed by SRM-MS to examine whether the biofilm and planktonic samples were differentially expressed. Data for both the “heavy” and “light” populations was collected for 54 of the targeted 61 peptides (Additional file 15). Figure 4, Table 2, and Additional file 16 show the data from 10 of the 19 proteins that we obtained consistent quantitative results to those observed in our initial Protein Pilot analysis of the MS/MS data. The 9 remaining proteins had data that was either inconclusive or was inconsistent with our initial MS/MS dataset and thus were not confirmed by SRM analyses. Of the 10 proteins that were confirmed by SRM analyses, four showed downregulation and six showed upregulation in the biofilm samples compared to the planktonic samples, with all six of the upregulated proteins showing an average peptide ratio of >1.5 and one of the downregulated proteins showing an average peptide ratio of <0.66. The cysteinyl-tRNA synthetase protein, aerobic respiration control protein ArcA, the predicted regulator of cell mophogenesis and NO signaling protein, and the molybdate-binding periplasmic protein were all seen as downregulated in the biofilm. The NAD nucleotidase protein, the heme-binding protein A, the glutamine synthetase protein, the protective surface antigen D15 protein, the probable acyl carrier protein phosphodiesterase, and the DNA gyrase protein subunit A were all found by SRM to be upregulated in the biofilm. One to five distinct peptides were used to target each protein in our multiplexed SRM-MS assays (Additional file 16). Of all of the proteins quantified by SRM, the aerobic respiration control protein ArcA showed the highest level of downregulation in the biofilm and the NAD nucleotidase protein and the heme-binding protein A showed the highest levels of upregulation in the biofilm.

**Table 2:** **Proteins shown by SRM to be have increased or decreased expression in the biofilm**

^**1**^ **Access. #**	**Protein**	^**2**^ **B:P**
AAX87080.1	cysteinyl-tRNA synthetase	0.78
AAX87923.1	aerobic respiration control protein ArcA	0.39
AAX88729.1	predicted regulator of cell morphogenesis and NO signaling	0.74
AAX88749.1	molybdate-binding periplasmic protein	0.93
**AAX87263.1**	**NAD nucleotidase**	**3.11**
**AAX87899.1**	**heme-binding protein A**	**1.85**
**AAX87912.1**	**glutamine synthetase**	**2.05**
**AAX87955.1**	**protective surface antigen D15**	**1.70**
**AAX88571.1**	**probable acyl carrier protein phosphodiesterase**	**2.75**
**AAX88658.1**	**DNA gyrase subunit A**	**2.24**

^1^Non-bold proteins were found to have decreased expression in the biofilm, proteins shown in bold were found to have increased expression in the biofilm.

^2^Average of all of the peptide ratios of the biofilm (B): planktonic (P) observed for the designated protein.

## Discussion

In the current study, heavy-labeled isoleucine was metabolically incorporated into the biofilm of the isoleucine auxotroph, *H. influenzae*. Unlabeled planktonic organisms were mixed with those from the heavy-labeled biofilm cultures, three protein extracts were generated, digested with trypsin and analyzed by ESI-MS/MS. Database searches showed that a number of proteins had differential expression in the biofilm versus planktonic samples. A select group of these proteins was further evaluated by SRM-MS to verify our initial findings. The work presented here is the first quantitative comparison between the proteomes of *H. influenzae* biofilm and planktonic organisms using SILAC metabolic labeling. While there have been some previous proteomic studies of *H. influenzae*, these were limited to the identification of proteins in the extracellular matrix of biofilm populations (42) or to bacteria grown in human sputum [46]. In this latter study, Qu *et al.* identified thirty-one proteins with expression level ratios >1.5 in the sputum samples compared to organisms grown in defined media [46], many of these differentially expressed proteins were involved in stress response, anti-oxidant response, nutrient uptake, and adherence.

In the current study, SILAC labeling was used to compare the global proteomic profiles of planktonic and biofilm samples of *H. influenzae* to gain insight on how the organism remodels its proteome for these diverse growth conditions. This labeling strategy incorporated a heavy labeled amino acid metabolically into the biofilm population and allowed us to directly differentiate between the biofilm and planktonic proteins due to a mass shift in their corresponding peptides. The ability to differentiate between the two populations allowed the samples to be combined early in the sample work-flow, thus reducing variability that could arise from sample processing. Additionally, unlike the previous studies which utilized a single biological replicate (42, 43), we compared the biofilm and planktonic proteomes of three biological replicates. Using this approach, we identified 814 unique proteins with 99% confidence that had a minimum of two peptides. Of these proteins, 127 showed variable expression between the biofilm and planktonic samples, with 26 proteins showing a protein abundance change of >1.5 and 39 proteins showing a protein abundance change of <0.66 in at least one biological replicate. Of the differentially expressed proteins, biological replicates #1 and #3 showed good concordance with 23 proteins having the same trend in both replicates; while biological replicate #2 did not correlate as well with the other two replicates, with 4 proteins showing similar trends in both cases. The overall number of proteins identified in biological replicate #2 (731 proteins) corresponded well with replicates #1 and #3 (737 and 684 proteins, respectively). In addition, the bias factor, which represents how well the mix of the labeled and unlabeled samples are mixed, was 1.22 for biological replicate #2 compared to 0.99 and 1.02 for biological replicates #1 and #3, respectively. The similarity in the overall number of proteins identified and the bias factors for replicate #2 compared to replicates #1 and #3 suggest that the differences seen in these replicates are not due to sample preparation. We believe the most likely explanation for the differences in the biofilm proteomic analyses of biological replicates #1 and #3 compared to replicate #2 is due to phase variation of one or more genes.

Phase variation is described as a random process by which a clonal population of microbes can present heterogeneous phenotypes as a result of a reversible genetic event [47,48]. The process can involve several mechanisms, including slipped-strand mispairing (SSM), site-specific recombination, and epigenetic regulation mediated by DNA methylation [47]. SSM is a mechanism found in human pathogens, including pathogenic *Neisseria*, *Bordetella pertussis*, *H. influenzae*, and *Helicobacter pylori*. The resulting frame shift causes on-off changes in gene expression [49].

Among phase variable genes in NT*Hi*, phase variation can occur every 5 × 10^−3^ to 2 × 10^−4^ colony forming units depending on the length of the polynucleotide repeat [49] and if this variant positively affects fitness it can become a predominant member of the population [50]. Thus, when single colonies are selected for in studies, these variants can be inadvertently selected for, thus biasing results. The sequenced NT*Hi* genomes have at least 13 virulence related genes containing long nucleotide repeat sequences either within the open reading frames themselves or within promoter regions of genes [49-51]. During transcription of these long repeat regions by DNA polymerase loss or gain of a repeat unit results in putting the gene out of frame with premature termination of transcription [47,48]. Analysis of strain comparison data within a NT*Hi* strain can be complicated by the fact that virulence-related phenotypes such as LOS structure, iron uptake, fimbriae and surface proteins are often subject to phase variation and these genes are important in biofilm formation [48]. As each of 13 or more phase-variable genes in each strain switches on and off in an independent fashion a single culture of NT*Hi* might consist of a mixture of hundreds of different variants that differ in expression of a number of factors important in biofilm formation [2].

Previous studies have shown that growth within a biofilm is highly complex, with both actively growing cells as well as inactive cells depending on the position within the biofilm and the overall state of the biofilm [52]. Dispersal of the biofilm at various time-points has also been observed and most likely arises due to a number of factors such as nutrient depletion or the action of small molecules [53]. Also, despite the fact that *in vitro* biofilms appear to form distinct structures, evidence indicates that biofilm forming organisms do not possess comprehensive genetic programs for biofilm development [54]. Whether the variability seen in biological replicate #2 is due to the biofilm undergoing changes due to phase variation, environmental factors or dispersal is unknown. It is clear however, that when the proteins that showed variable expression in any of the replicates were categorized by function and plotted (Figure 3), a small set of pathways seemed to show the most change. These groups, which showed the most changes, include proteins involved in DNA metabolism, energy metabolism and protein synthesis.

Proteins involved in DNA metabolism showed a general trend towards increased expression in the biofilm. These proteins include the DNA mismatch repair protein MutS, a transcription-repair coupling factor, protein A from the UvrABC system and the DNA-binding protein HU, amongst others. All of these proteins have been implicated in either DNA repair or in protection against denaturation under environmental stress. It has been predicted that *H. influenzae* within a biofilm may be under oxidative stress, and this could cause DNA damage. In a microarray study comparing biofilm and planktonic cultures of NT*Hi* by Pang and co-workers, transcripts for a number of factors involved in bacterial stress-response were reported to be upregulated in the biofilms [55]. One of these factors was a homolog of the DNA-binding protein associated with starvation (Dps). A *dps* mutant was generated in NT*Hi*, and this mutant showed increased susceptibility to environmental stress and had reduced survival within a biofilm compared to the parent strain [55]. In a separate study, Gawnorski *et al.* used a genomic approach to investigate genes important in *H. influenzae* pathogenesis and also found that genes involved in DNA repair and oxidative stress were important for virulence in a murine pulmonary infection model [56]. In their study, they used a methodology designated HITS for ‘high-throughput insertion tracking by deep sequencing’. In this approach, transposon insertion libraries were generated and then sequenced either before or after passage in the murine lung model; the sequence outputs from before and after passage were then compared to determine genes important in pathogenesis. Their studies showed that the DNA repair genes *ruvA*, *ruvB*, *recR*, *recC*, *xerC* and *xerD* were all found to be necessary for virulence [56]. These studies also showed that the oxidative stress response genes *pgdX* and *oxyR* were necessary for a murine lung infection. Murphy *et al.* showed that the anti-oxidant enzyme, peroxiredoxin/glutaredoxin glutathione dependent peroxidase (PgdX), was expressed at higher levels in static biofilms compared to planktonic cultures [57]. This study also showed that COPD patient sera post-exacerbation had higher PgdX levels compared to paired patient sera pre-exacerbation [57]. The levels of PdgX were also shown to be elevated in *H. influenzae* grown in a sputum culture when compared to bacteria grown in a chemically defined media [46]. Unlike these previous studies, significant differences in PdgX levels of the biofilm compared to the planktonic bacteria were not detected in our study. This difference could be due to the growth state of our biofilm compared to the growth state of the samples from the previous studies. It is clear, however, that both in our study and in previous studies that the upregulation of DNA repair mechanisms is important for *H. influenzae* to cope with oxidative stress.

Energy metabolism and protein synthesis proteins were generally expressed at lower levels in the *H. influenzae* biofilm samples compared to the planktonic organisms. Although there is metabolic heterogeneity within the biofilm, reduced metabolism in bacterial biofilms is a generally accepted phenomenon [14,58]. The shift to a stationary or dormant phase within the biofilm seems to play an important role in antibiotic resistance [14,58,59]. For example, when Fux and colleagues evaluated *Staphylococcus aureus* biofilm “clumps”, which had detached from the biofilm, they found that they were highly resistant to the antibiotic oxacillin [60]. This level of resistance was similar to stationary phase planktonic organisms grown in spent medium, suggesting a direct connection between metabolic activity and antibiotic resistance [60]. In a similar study, Anderl *et al.* showed that even though both ampicillin and ciprofloxacin were able to penetrate a *Klebsiella pneumoniae* biofilm, the organisms remained resistant to clearing by these drugs [61]. Planktonic bacteria, from stationary phase, grown in media lacking carbon and nitrogen sources, were similarly antibiotic resistant as the biofilm organisms [61]. When biofilm bacteria were dispersed into rich media they once again became susceptible to antibiotics [61]. These studies, as well as others, clearly demonstrate that reduced metabolism is not only a necessity due to nutrient limitation conditions, but that it is also likely a survival mechanism that allows the biofilm to escape clearance by antimicrobials. Our findings that both protein synthesis and metabolism decrease in the *H. influenzae* biofilm further strengthen the importance of these survival mechanisms.

The aerobic respiration control protein ArcA was found to be the most highly downregulated protein in our study. Our initial screen found that the biofilm:planktonic expression ratio of ArcA in two biological replicates was less than 0.37. These results were further confirmed in our SRM studies, where two peptides were found to have an average biofilm:planktonic ratio of 0.39. *H. influenzae* encounters various levels of oxygen conditions both within the host and within biofilms; therefore, the bacteria has devised various mechanisms to cope with differing levels of oxygen availability. One such mechanism is ArcA [62-64]. This protein is part of a two-component regulatory system (ArcAB) that has been previously shown to be involved in modulating genes needed for adaptation to changes in respiration [62-64]. ArcA is thought to be most active under low oxygen conditions [63,64]. In *E. coli* ArcAB regulate genes involved in metabolic pathways, such as the TCA cycle, in response to respiratory conditions [65]. Similarly, ArcA was also found to modulate the expression level of genes involved in metabolic pathways in *H. influenzae* [64]. A recent metabolomics analysis of *H. influenzae* showed that the only TCA cycle enzymes present in *H. influenzae* are malate dehydrogenase, fumarate hydratase, succinyl CoA synthetase, and α-ketoglutarate dehydrogenase [66]. Of these, both malate dehydrogenase and fumarate hydratase were found in our study to be downregulated in the biofilm. As Table 1 demonstrates, a number of proteins involved in respiration were also shown to be decreased in expression, including PfkA, FrdA, Fba, Eno, and PykA. Conversely, some proteins involved in this functional group were shown to be increased in expression, such as CitF, NsfB, Mao2, and Dld.

Biofilms are complex entities with evidence of stratified growth within their internal structures. One reason for this type of growth is in part due to the varying oxygen concentrations within a biofilm. Oxygen levels are higher near the surface, while oxygen levels near the base of the biofilm are most likely low. Indeed, Werner and co-workers used oxygen electrodes to show that oxygen only penetrated approximately 50 μm into the biofilm [52]. These variations in oxygen levels within biofilms make respiration within a biofilm complex. Our data combined with previous studies suggest that some sections of the strata function at an aerobic level, while other sections of the biofilm operate in either microaerophilic or anaerobic environments. Adding to this complexity, ArcA was recently shown to regulate *dps* [64]. Dps has been shown to be important in dealing with oxidative stress resistance [55,64]. Since ArcA seems to be most active in low oxygen conditions, but paradoxically modulates genes important in coping with oxidative stress, it seems likely that ArcA and Dps may help the bacteria shift between low and high oxygen conditions.

The NAD nucleotidase protein NadN was found at higher levels in the biofilm organisms compared to the planktonic bacteria in all three biological replicates. SRM data further confirmed this with an average biofilm:planktonic ratio of 3:1. This ratio was determined from 4 peptides which were observed in all three biological replicates and one peptide that was observed in two biological replicates. The combination of the difference between the biofilm and planktonic levels being consistently observed amongst all three biological replicates as well as the high level of difference between NadN levels in the biofilm and planktonic conditions suggests that this protein is crucial in *H. influenzae* biofilm formation. It is known that *H. influenzae* lack most of the enzymes necessary for *de novo* synthesis of NAD; therefore, these bacteria have an absolute requirement for exogenous NAD (factor V). Previous studies have demonstrated that NadN is a periplasmic protein which functions as both an NAD pyrophosphatase and an NMN 5’-nucleotidase, and is necessary for growth on NAD *in vitro* [67,68]. Gawronski *et al.* showed that *nadN* was crucial for *H. influenzae* infection in a murine lung infection model [56]. Their studies also demonstrated the necessity of *hel*, which is also involved in NAD utilization, in their infection model [56]. It is clear that NAD utilization is crucial under a number of growth conditions. The increase in NadN levels in *H. influenzae* biofilms suggests that uptake and utilization of NAD is important in biofilm formation and/or maintenance.

In addition to *H. influenzae’s* requirement of NAD for growth, these organisms also require heme. *H. influenzae* have developed a number of mechanisms for acquiring heme from the human host including heme binding proteins and heme utilization systems (for example: [69-77]). HbpA, was originally identified as being a heme binding protein [71,75,78], but has subsequently been asserted to be primarily involved in glutathione acquisition [79]. The first identification of *H. influenzae* HbpA was achieved through transformation of an *H. influenzae* library into *E. coli* followed by screening for heme binding activity [78]. Morton *et al.* later showed that HbpA bound various forms of heme and *hbpA* mutants were unable to bind heme [75]. Morton and colleagues also showed that an *hbpA* mutant in a *H. influenzae* type B strain was reduced in its virulence in a 30 day old rat model of bacteremia [80]. More recently, Vergauwen and colleagues demonstrated that HbpA bound both reduced glutathione (GSH) and oxidized glutathione (GSSG) at physiologically relevant levels, while it only weakly bound hemin [79]. They proposed that glutathione is transported across the bacterial inner-membrane using an ATP-binding cassette (ABC)-like dipeptide transporter (DppBCDF), with HbpA as the periplasmic binding protein [79]. This group proposed to change the nomenclature of this protein from HbpA to GbpA (glutathione binding protein) [79]. In the present study, HbpA was found to be expressed at higher levels in the biofilm organisms compared to the planktonic bacteria. The reduced form of glutathione (GSH) is able to aid in the reduction of reactive oxygen species. If HbpA is truly a glutathione transporter, able to transport both GSH and GSSG, it is feasible that this protein may be important in assisting *H. influenzae* biofilm organisms with oxidative stress conditions. Conversely, if HbpA is primarily involved in acquiring heme, this protein most likely plays an important role in helping the biofilm organisms acquire iron from the host. Whether HbpA’s primary role is binding heme, binding glutathione, or perhaps both, it is interesting to hypothesize that the promiscuity of HbpA’s substrate binding may be by necessity or design and could play an important role in *H. influenzae* biofilm growth and virulence.

## Conclusions

It is clear from the current study, as well as previous studies, that some common factors are important in biofilm formation. *H. influenzae* within a biofilm need ways of dealing with the various effects of oxidative stress and damage. The ability to obtain necessary growth factors, such as NAD and heme, are also crucial for survival. Biofilm organisms exist in a somewhat dormant state with reduced energy metabolism and reduced protein synthesis. It still remains unclear what bacterial factors, if any, regulate *H. influenzae* biofilm formation and maintenance. In the present study, we employed a SILAC metabolic labeling strategy to quantitatively compare the proteomes of the biofilm and planktonic states of NT*Hi*. One of the major advantages of this approach over unlabeled strategies is the ability of the samples to be mixed early on in the processing steps. The key disadvantage of a comparative strategy is that it is not able to measure the absence of a protein in one condition compared to the other condition. Future strategies for evaluating the proteomes of these two growth conditions may be able to address this issue. In addition, large scale proteomic approaches typically miss comparisons of peptides with post-translational modifications (PTMs), as these peptides are usually a small percentage of the total peptide population. With the recent discoveries of a number of PTMs in bacteria [81], it seems reasonable to assume that PTMs will play at least some role in biofilm formation or maintenance. The data presented here, coupled with future studies, should provide a good foundation for potential drug therapy targets that could lead to more effective clearance of resistant NT*Hi* biofilm organisms.

## Additional files

Additional file 1:
**Total unique proteins 99% Conf and 2 peptides with 95% conf.**
Additional file 2:
**Proteins identified with at least 99% confidence and at least two peptides with 95% confidence in Replicate #1.**
Additional file 3:
**Proteins differentially expressed, with p-values ≤ 0.05 and EF <2, in Replicate #1.**
Additional file 4:
**Protein Pilot peptide summary of peptides used for quantitation of proteins whose p value for H:L ratio was ≤ 0.05 in Replicate #1.**
Additional file 5:
**Proteins Identified with at least 99% Confidence and at least two peptides at 95% confidence in Replicate #2.**
Additional file 6:
**Proteins differentially expressed, with p-values ≤ 0.05 and EF <2, in Replicate #2.**
Additional file 7:
**Protein Pilot peptide summary of peptides used for quantitation of proteins whose p value for H:L ratio was ≤ 0.05 for Replicate #2.**
Additional file 8:
**Proteins Identified with at least 99% Confidence and at least two peptides at 95% confidence in Replicate #3.**
Additional file 9:
**Proteins differentially expressed, with p-values ≤ 0.05 and EF <2, in Replicate #3.**
Additional file 10:
**Protein Pilot peptide summary of peptides used for quantitation of proteins whose p value for H:L ratio was ≤ 0.05 for Replicate #3.**
Additional file 11:
**Proteins predicted, by Protein Pilot, to have variable up- or downregulation in the biofilm.**
Additional file 12:
**The genes encoding proteins involved in pyruvate metabolism that were found to be differentially expressed in the biofilm:planktonic samples were plotted on KEGG pathways.**
Additional file 13:
**The genes encoding proteins involved in glycolysis that were found to be differentially expressed in the biofilm:planktonic samples were plotted on KEGG pathways.**
Additional file 14:
**The genes encoding proteins involved in the pentose phosphate pathway that were found to be differentially expressed in the biofilm:planktonic samples were plotted on KEGG pathways.**
Additional file 15:
**Peptides targeted for SRM analyses.**
Additional file 16:
**Proteins shown by SRM to be up- or downregulated.**

## Abbreviations

NT*Hi*: Non-typeable *Haemophilus influenzae*
SILAC: Stable isotope labeling by amino acids in cell culture
LC-MS: Liquid chromatography-mass spectrometry
SRM-MS: Selected reaction monitoring-mass spectrometry
COPD: Chronic obstructive pulmonary disease
LOS: Lipooligosaccharide
MALDI-TOF: Matrix-assisted laser desorption ionization time-of-flight
ESI-MS: Electrospray ionization-mass spectrometry
