# Supplementary material for: Comparative analyses of proteins from Haemophilus influenzae biofilm and planktonic populations using metabolic labeling and mass spectrometry
Source: BMC Microbiol. 2014 Dec 31;14:329. doi: 10.1186/s12866-014-0329-9 (PMC4302520; doi:10.1186/s12866-014-0329-9)
Supplement: Additional file 11: — Proteins predicted, by Protein Pilot, to have variable up- or downregulation in the biofilm. [file 12866_2014_329_MOESM11_ESM.docx]

**Additional Table 11.** Proteins predicted, by Protein Pilot, to have variable up- or downregulation in the biofilm.

| gi # | Accession # | Protein Name | B:P ratio Rep. 1 | B:P ratio Rep. 2 | B:P ratio Rep. 3 |
| --- | --- | --- | --- | --- | --- |
| gi\|68056968 | AAX87221.1 | glutathione reductase |  | 1.29 | 0.66 |
| gi\|68057096 | AAX87349.1 | transcription termination factor rho | 0.66 |  | 1.89 |
| gi\|68057584 | AAX87837.1 | 50S ribosomal protein L24 | 0.55 |  | 1.28 |
| gi\|68058244 | AAX88497.1 | universal stress protein E | 0.79 | 1.35 |  |
| gi\|68058536 | AAX88789.1 | L-lactate dehydrogenase |  | 0.81 | 1.25 |
