# Supplementary material for: Comparative analyses of proteins from Haemophilus influenzae biofilm and planktonic populations using metabolic labeling and mass spectrometry
Source: BMC Microbiol. 2014 Dec 31;14:329. doi: 10.1186/s12866-014-0329-9 (PMC4302520; doi:10.1186/s12866-014-0329-9)
Supplement: Additional file 12: — The genes encoding proteins involved in pyruvate metabolism that were found to be differentially expressed in the biofilm:planktonic samples were plotted on KEGG pathways. [file 12866_2014_329_MOESM12_ESM.pptx]

## Slide 1
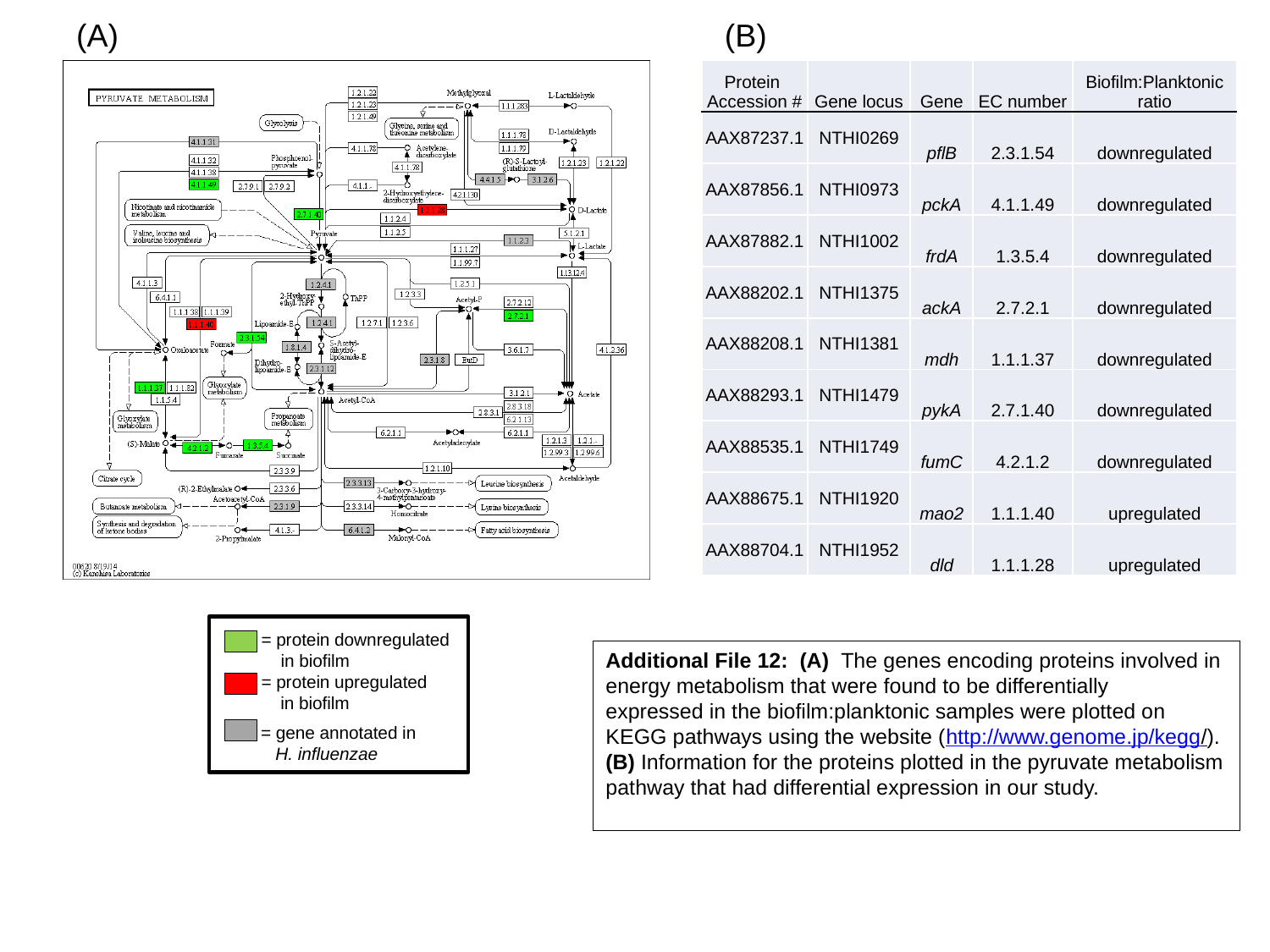

(A)
(B)
| Protein Accession # | Gene locus | Gene | EC number | Biofilm:Planktonic ratio |
| --- | --- | --- | --- | --- |
| AAX87237.1 | NTHI0269 | pflB | 2.3.1.54 | downregulated |
| AAX87856.1 | NTHI0973 | pckA | 4.1.1.49 | downregulated |
| AAX87882.1 | NTHI1002 | frdA | 1.3.5.4 | downregulated |
| AAX88202.1 | NTHI1375 | ackA | 2.7.2.1 | downregulated |
| AAX88208.1 | NTHI1381 | mdh | 1.1.1.37 | downregulated |
| AAX88293.1 | NTHI1479 | pykA | 2.7.1.40 | downregulated |
| AAX88535.1 | NTHI1749 | fumC | 4.2.1.2 | downregulated |
| AAX88675.1 | NTHI1920 | mao2 | 1.1.1.40 | upregulated |
| AAX88704.1 | NTHI1952 | dld | 1.1.1.28 | upregulated |
= protein downregulated
 in biofilm
= protein upregulated
 in biofilm
= gene annotated in
 H. influenzae
Additional File 12: (A) The genes encoding proteins involved in
energy metabolism that were found to be differentially
expressed in the biofilm:planktonic samples were plotted on
KEGG pathways using the website (http://www.genome.jp/kegg/).
(B) Information for the proteins plotted in the pyruvate metabolism
pathway that had differential expression in our study.
