# Supplementary material for: Comparative analyses of proteins from Haemophilus influenzae biofilm and planktonic populations using metabolic labeling and mass spectrometry
Source: BMC Microbiol. 2014 Dec 31;14:329. doi: 10.1186/s12866-014-0329-9 (PMC4302520; doi:10.1186/s12866-014-0329-9)
Supplement: Additional file 14: — The genes encoding proteins involved in the pentose phosphate pathway that were found to be differentially expressed in the biofilm:planktonic samples were plotted on KEGG pathways. [file 12866_2014_329_MOESM14_ESM.pptx]

## Slide 1
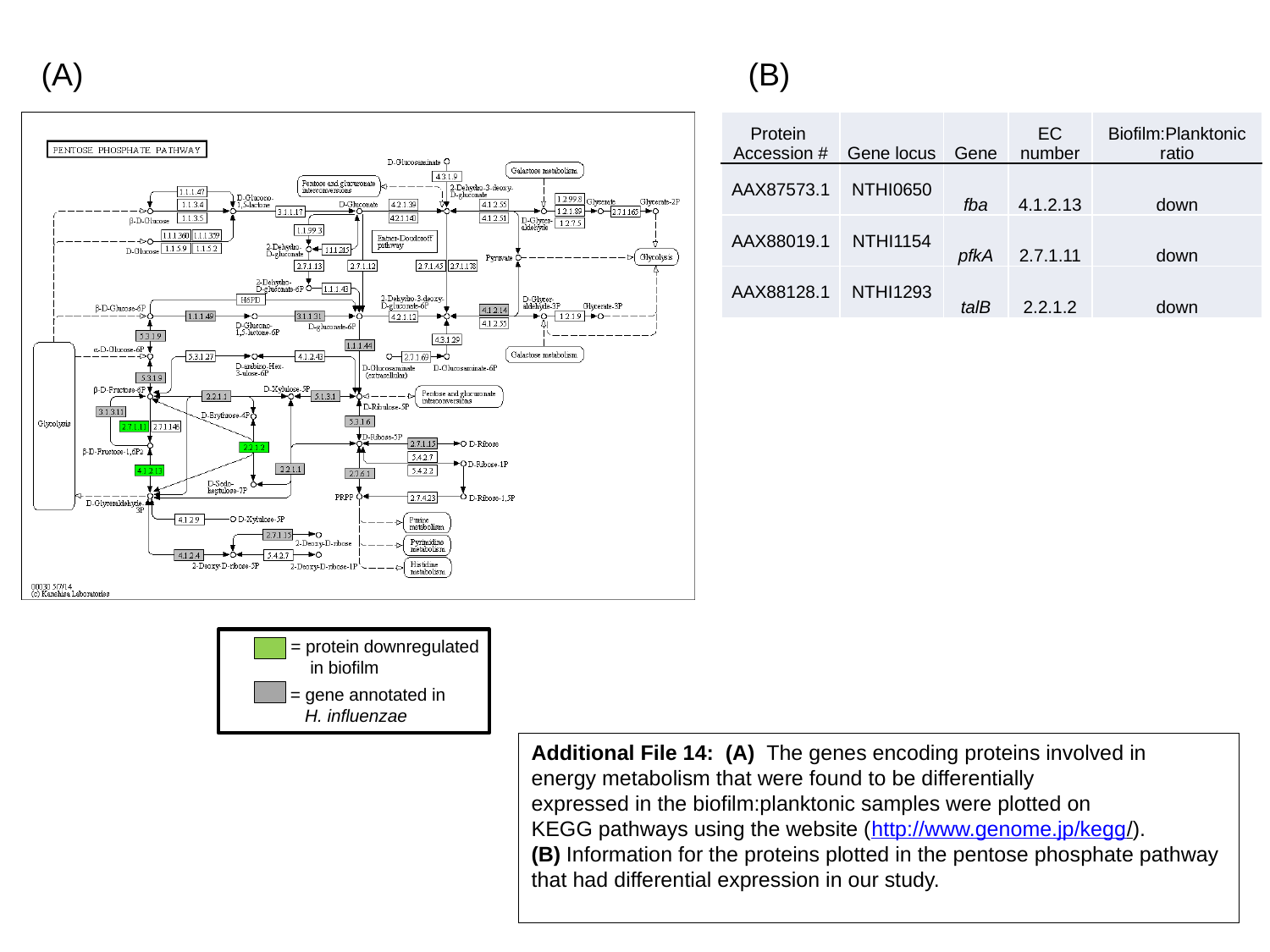

(A)
(B)
| Protein Accession # | Gene locus | Gene | EC number | Biofilm:Planktonic ratio |
| --- | --- | --- | --- | --- |
| AAX87573.1 | NTHI0650 | fba | 4.1.2.13 | down |
| AAX88019.1 | NTHI1154 | pfkA | 2.7.1.11 | down |
| AAX88128.1 | NTHI1293 | talB | 2.2.1.2 | down |
= protein downregulated
 in biofilm
= gene annotated in
 H. influenzae
Additional File 14: (A) The genes encoding proteins involved in
energy metabolism that were found to be differentially
expressed in the biofilm:planktonic samples were plotted on
KEGG pathways using the website (http://www.genome.jp/kegg/).
(B) Information for the proteins plotted in the pentose phosphate pathway
that had differential expression in our study.
