# Supplementary material for: Comparative analyses of proteins from Haemophilus influenzae biofilm and planktonic populations using metabolic labeling and mass spectrometry
Source: BMC Microbiol. 2014 Dec 31;14:329. doi: 10.1186/s12866-014-0329-9 (PMC4302520; doi:10.1186/s12866-014-0329-9)
Supplement: Additional file 16: — Proteins shown by SRM to be up- or downregulated. [file 12866_2014_329_MOESM16_ESM.docx]

| **Additional Table 16.** Proteins shown by SRM to be up- or downregulated. | | |  |  |  |
| --- | --- | --- | --- | --- | --- |
|  |  |  |  |  |  |
| **^1^Accession #** | **Protein** | **Peptide** | **^2^B:P** | **# replicates** | **std dev** |
| AAX87080.1 | cysteinyl-tRNA synthetase | ELGAILGLLQQDPEK | 0.755 | 2 | 0.138 |
|  |  | SLGNFFTIR | 0.802 | 2 | 0.210 |
| AAX87923.1 | aerobic respiration control protein ArcA | ELREELSLPLIFLTGR | 0.299 | 1 | N/A |
|  |  | LDLNSHSLITPEGQEFK | 0.516 | 1 | N/A |
| AAX88729.1 | predicted regulator of cell morphogenesis and NO signaling | EQLPELITLAEK | 0.665 | 3 | 0.232 |
|  |  | LSELAVSIPGATK | 0.819 | 3 | 0.309 |
| AAX88749.1 | molybdate-binding periplasmic protein | AEAPYGIVYSTDAK | 0.886 | 3 | 0.171 |
|  |  | SAVNSVDIAK | 0.922 | 3 | 0.188 |
|  |  | TVAVFPADSHKPVVYPVSIVK | 0.945 | 3 | 0.231 |
|  |  | VLVGNDLVLIAPAK | 0.949 | 3 | 0.210 |
| AAX87263.1 | NAD nucleotidase | IAIIGLDTVNK | 3.291 | 3 | 1.581 |
|  |  | IILLSHAGSEK | 4.001 | 3 | 2.015 |
|  |  | LPVIYEYPLEFK | 3.667 | 3 | 2.725 |
|  |  | TVDLTIQNAGGVR | 2.526 | 3 | 0.838 |
|  |  | WKPYDIFTVDGEK | 2.382 | 2 | 0.514 |
| AAX87899.1 | heme-binding protein A | FNNSEFDALLNEAIGLTNKEER | 3.317 | 1 | N/A |
|  |  | GLNVAYIAFNTEK | 1.757 | 2 | 0 |
|  |  | LVISIVPDATTR | 2.138 | 3 | 0.935 |
|  |  | TDQAIQYVAHENYWK | 0.943 | 2 | 0.772 |
| AAX87912.1 | glutamine synthetase | IPAVTNPK | 2.046 | 3 | 0.511 |
| AAX87955.1 | protective surface antigen D15 | ISNFALEYNR | 1.699 | 3 | 0.632 |
|  |  | YNATVEPIVNTLPNNR | 1.700 | 3 | 0.809 |
| AAX88571.1 | probable acyl carrier protein phosphodiesterase | LQGNNIVVR | 1.957 | 2 | 1.085 |
|  |  | SSISGNNSQTNQLADYVIEK | 2.261 | 1 | N/A |
|  |  | SYFDFIARPR | 4.709 | 1 | N/A |
| AAX88658.1 | DNA gyrase subunit A | GRPIVNILPLQENER | 1.841 | 3 | 0.819 |
|  |  | IADLLHILSSAER | 1.448 | 3 | 0.626 |
|  |  | THILEGLAVAR | 6.165 | 3 | 4.445 |
|  |  | VVGDVIGK | 2.035 | 3 | 0.772 |
|  |  | YHPHGDSAVYDTIVR | 1.693 | 3 | 0.233 |
| ^1^Unshaded proteins were found to be downregulated in the biofilm, shaded proteins were found to be upregulated in the biofilm.  **^2^**Biofilm:Planktonic or “Heavy” (H): “Light” (L) | | | | |  |
